# Navigating hypertriglyceridemia in pregnancy: current evidence and clinical strategies

**DOI:** 10.3389/fendo.2026.1761761

**Published:** 2026-05-08

**Authors:** Szymon Chamerski, Katarzyna Wartecka-Zielińska, Filip Pomorski, Wiktoria Karos, Joanna Marlęga-Linert, Marta Marcinkowska, Katarzyna Stefańska, Magdalena Grzybowska, Dariusz Wydra, Marcin Gruchała, Agnieszka Mickiewicz

**Affiliations:** 1 First Department of Cardiology, University Clinical Center, Medical University of Gdańsk, Gdańsk, Poland; 2 Department of Emergency Medicine, University Clinical Center, Medical University of Gdańsk, Gdańsk, Poland; 3 Department of Gynaecology and Obstetrics, University Clinical Center, Medical University of Gdańsk, Gdańsk, Poland

**Keywords:** hypertriglyceridemia, lipid disorders, preeclampsia, pregnancy, strategy

## Abstract

Pregnancy induces profound metabolic adaptations, including marked rises in lipid concentrations. Triglyceride levels may increase by 100–300% compared with pre-pregnancy values, and in some women this physiological response exceeds the adaptive range. Although triglyceride elevation alone does not necessarily increase the risk of complications, higher triglyceride levels have been linked to maternal disorders such as preeclampsia, gestational hypertension, and acute pancreatitis, as well as adverse fetal outcomes, including abnormal birth weight and preterm delivery. Management of hypertriglyceridemia in pregnancy is challenging due to limited pharmacologic options and the need to ensure fetal safety. A personalized, multidisciplinary strategy—based on a low-fat diet, weight optimization, and regular physical activity—remains first-line therapy. In severe hypertriglyceridemia with imminent pancreatitis risk, rapid interventions such as pharmacotherapy or therapeutic plasma exchange are required to promptly reduce triglyceride levels. Novel agents targeting apolipoprotein C-III (olezarsen, volanesorsen) can lower triglycerides by 40–70%, but their safety in pregnancy is not established. While olezarsen is not recommended, two case reports describe successful and safe use of volanesorsen in pregnant women with familial chylomicronemia syndrome under close monitoring. This narrative review synthesizes current evidence on the pathophysiology, prognostic implications, and management of hypertriglyceridemia in pregnancy, emphasizing early risk identification, first-trimester lipid assessment, and individualized treatment aligned with the updated ESC/EAS guidelines.

## 1 Introduction

Pregnancy represents a unique period during which maternal metabolism undergoes extensive adaptations to provide optimal conditions for fetal and placental development. Among these changes, lipid metabolism is significantly altered, with a physiological increase in maternal blood lipid concentrations, particularly during the second and third trimesters, to supply the fetus with sufficient lipid substrates for energy, structural components, and signaling functions (1).

Triglycerides (TG) are especially susceptible to elevating during pregnancy, with levels potentially increasing by 150–300% compared to the pre-pregnancy period (2, 3). In some women, this adaptive increase may exceed physiological limits and acquire pathological characteristics. Hypertriglyceridemia (hyperTG) in pregnancy has been associated with numerous maternal and fetal complications, including preeclampsia, pregnancy-induced hypertension, preterm delivery, abnormal fetal birth weight, and acute pancreatitis—a severe condition requiring urgent intervention (4).

It is important to note that not all cases of hyperTG observed during pregnancy result from maladaptive metabolic changes. Abnormal lipid profiles may preexist due to obesity, diabetes, metabolic syndrome, or, less commonly, genetically determined lipid metabolism disorders (5, 6). Increasing evidence suggests that early lipid abnormalities, detectable in the first trimester, may serve as predictive markers for both obstetric complications and future cardiovascular disease (4, 7).

The management of hyperTG in pregnant women remains challenging due to limited pharmacological options, the need to ensure fetal safety, and the lack of comprehensive guidelines stemming from insufficient data. Standard approaches primarily rely on lifestyle interventions, including dietary modification, weight management, and increased physical activity. Pharmacotherapy is reserved for carefully selected cases after thorough evaluation of risks and benefits. In instances of acute pancreatitis, insulin and heparin administration, as well as hospital-based plasmapheresis, may be necessary (8, 9).

This review aims to summarize current research on hyperTG in pregnancy, evaluate its impact on maternal and fetal outcomes, and provide an overview of available therapeutic strategies.

## 2 Triglyceride metabolism in pregnancy

During pregnancy, the maternal organism undergoes multiple metabolic adaptations to support proper fetal development. These changes are particularly pronounced in lipid metabolism, especially regarding triglycerides (TG). Maternal adaptations occur in two distinct phases.

During the first two-thirds of pregnancy, the maternal state is predominantly anabolic. Peripheral tissue insulin sensitivity increases, partly through upregulation of insulin receptor density. This, combined with heightened appetite and food intake (hyperphagia) and enhanced lipoprotein lipase (LPL) activity, promotes the synthesis and storage of TG in adipocytes, thereby ensuring energy reserves for the later stages of pregnancy and for lactation (10).

In the third trimester, maternal metabolism shifts to a catabolic state, characterized by increased insulin resistance and reduced LPL activity. Concurrently, lipolysis in adipose tissue intensifies, resulting in elevated levels of free fatty acids (FFAs) and glycerol in the maternal circulation. These substrates are taken up by the liver and used for TG synthesis, which are subsequently packaged into very-low-density lipoprotein (VLDL) particles and secreted into the bloodstream (10).

These metabolic adaptations, combined with reduced TG uptake by maternal adipose tissue, contribute to a 100–300% increase in maternal plasma TG levels compared with pre-pregnancy values (2, 3). Other lipid parameters also rise: total cholesterol (TC) and low-density lipoprotein cholesterol (LDL-C) increase by 30–50% (6, 11), high-density lipoprotein cholesterol (HDL-C) by 20–40% (11), and lipoprotein(a) (Lp(a)) by 100–200% (12–14). Lipid concentrations generally peak just before delivery, subsequently decline, and typically return to pre-pregnancy levels within approximately two months postpartum (15).

In some women, TG elevations exceed the physiological adaptive range and become pathological. Predisposing factors for pathological hyperTG in pregnancy include obesity, hypothyroidism, diabetes, insulin resistance, metabolic syndrome, and genetically determined lipid or lipoprotein disorders (15).

## 3 First-trimester triglyceride concentration as a predictor of pregnancy complications

An increasing number of studies indicate that early-pregnancy TG levels may serve not only as markers of metabolic adaptation but also as important predictors of obstetric complications.

Preeclampsia (PE) is a complex, multifactorial disorder occurring after the 20th week of gestation, defined by new-onset hypertension (systolic blood pressure ≥140 mmHg and/or diastolic blood pressure ≥90 mmHg) accompanied by proteinuria (≥0.3 g/24h) or other signs of organ dysfunction. Although the exact pathogenesis remains unclear, abnormal placental development, particularly impaired trophoblast invasion and insufficient spiral artery remodeling, plays a central role, resulting in chronic placental hypoxia, oxidative stress, immune dysregulation, and release of anti-angiogenic factors into the maternal circulation (16).

Elevated TG concentrations in early pregnancy are strongly associated with later PE development (17–19). According to a 2004 study by Enquobahrie et al., women who subsequently developed PE had 13.6% higher first-trimester TG levels compared to unaffected women (p < 0.05) (4). PE risk rises linearly with TG levels: at TG >133 mg/dL, the risk is 4.15-fold higher than in women with TG <93 mg/dL (adjusted OR 4.15, p = 0.005) (4). Other lipid abnormalities associated with increased PE risk include elevated total cholesterol (TC), LDL-C, LDL/HDL ratio, and reduced HDL-C (4). Additional maternal risk factors include advanced age, multiparity, elevated pre-pregnancy BMI, chronic renal, hepatic, and cardiac diseases, and pregestational hypertension.

**Figure 1:**
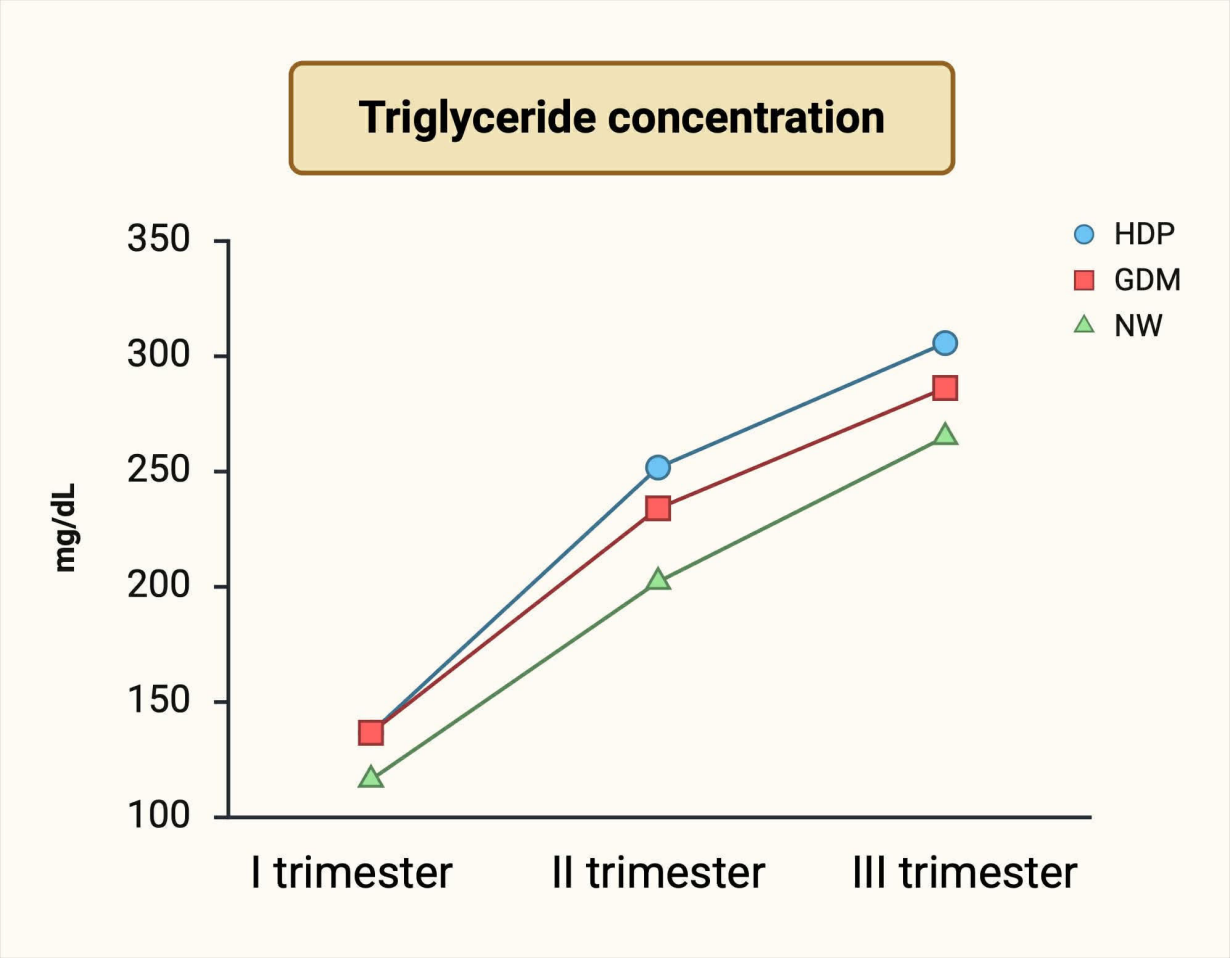
Changes in maternal triglyceride levels in different trimesters. Image created by the authors based on Shen et al. (2016) (19) under CC BY-NC 4.0. (NW, normal women; GDM, gestational diabetes mellitus; HDP, hypertensive disorders of pregnancy, including preeclampsia and gestational hypertension).

First-trimester TG levels have also been linearly associated with increased incidence of gestational hypertension (OR 1.60, p = 0.021), PE (OR 1.69, p = 0.018), large-for-gestational-age neonates (LGA) (OR 1.48, p = 0.001), and preterm birth (OR 1.69, p = 0.006) (7). High TG levels in the second trimester may predict early-onset PE (diagnosed before 34 weeks of gestation) (20). Li Juan et al. demonstrated that TG concentrations between 24 and 28 weeks are inversely correlated to the time of PE onset, with levels >229.4 mg/dL (>2.59 mmol/L)conferring a twofold increased risk of early-onset PE (20). Changes in maternal triglyceride concentrations in different trimesters have been presented in **Figure 1**.

**Figure 2:**
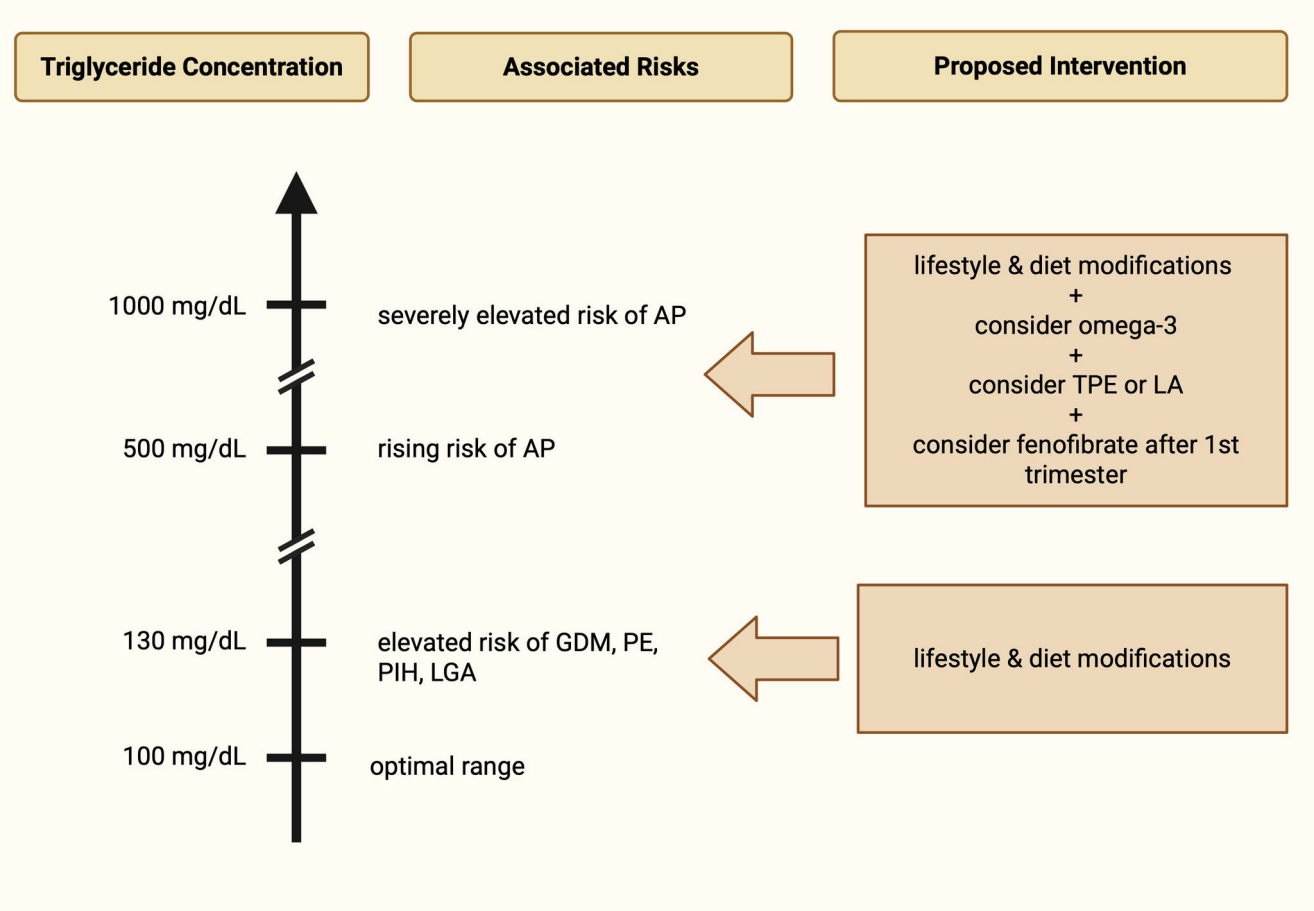
Risks associated with elevated triglycerides in pregnancy and proposed interventions (AP, acute pancreatitis; GDM, gestational diabetes mellitus; PE, preeclampsia; PIH, pregnancy-induced hypertension; LGA, large for gestational age; TPE, therapeutic plasma exchange; LA, lipoprotein apheresis).

Elevated early-pregnancy TG concentrations, together with ApoA, ApoB, TC, and LDL, are also associated with higher preterm birth risk (delivery <37 weeks), either as a complication of PE or independently (21). Although multiple risk factors for preterm birth have been identified, many cases remain unexplained, highlighting the importance of novel predictive markers such as TG, which are measurable already in early pregnancy (22). Preterm delivery has also been linked to a twofold higher long-term maternal cardiovascular disease (CVD) risk, including ischemic heart disease and stroke (23). This opens new opportunities for identifying women at higher long-term CVD risk and encouraging them—already early in pregnancy—to address modifiable risk factors such as diabetes, hypertension, dyslipidemia, and smoking. Risks associated with elevated triglycerides in pregnancy have been presented in **Figure 2**.

## 4 Role of triglycerides in metabolic syndrome and gestational diabetes

Metabolic syndrome (MetS) in pregnant women is characterized by obesity, insulin resistance, hypertension, and dyslipidemia, including elevated TG. A recent systematic review and meta-analysis by Mohebi et al. estimated that approximately 16% of pregnant women meet the criteria for MetS (24). Among these women, nearly half exhibited elevated triglycerides (48.5%) and obesity (42.7%), while high blood pressure was present in 37.7%, elevated fasting glucose in 16.2%, and low HDL cholesterol in 12.3%. The study also highlighted that the prevalence of MetS increases as pregnancy progresses, from 9.9% before 16 weeks to 24.1% after 20 weeks of gestation, emphasizing the importance of early screening and monitoring (24).

During pregnancy, circulating TG and free fatty acid (FFA) levels increase to support fetal growth. In women with metabolic syndrome MetS, these lipid adaptations can become exaggerated, leading to elevated accumulation of TG and non-esterified fatty acids (NEFA) in maternal tissues and the placenta. Placental fatty acid transport is mediated by fatty acid transport proteins (FATPs) and CD36, and is regulated by transcription factors such as peroxisome proliferator-activated receptor-γ (PPAR-γ) (25). Elevated NEFA levels during pregnancy have been shown to impair insulin signaling, contributing to insulin resistance and reducing glucose uptake in maternal tissues (26). Chronic exposure to high NEFA concentrations also promotes oxidative stress and β-cell dysfunction, contributing to impaired insulin secretion and apoptosis. Together, these disturbances underline the development of gestational diabetes mellitus (GDM) (27).

In a prospective cohort study by Enquobahrie et al., women with first-trimester TG concentrations ≥137 mg/dL had over a threefold higher risk of developing GDM compared with those with TG concentrations <96 mg/dL, and each additional 20 mg/dL increase in TG was associated with ~10% higher risk (28). Furthermore, analyses suggested that the combination of elevated TG and glucose levels may increase GDM risk up to sixfold (28).

## 5 Estrogen-induced hypertriglyceridemia in IVF

Assisted reproductive technologies, particularly *in vitro* fertilization (IVF), represent a clinically relevant setting in which supraphysiological estrogen exposure may significantly influence TG metabolism. Controlled ovarian stimulation leads to markedly elevated estradiol concentrations, which promote hepatic VLDL production, and reduce the clearance of TG from the circulation, resulting in higher serum TG levels. Although this effect is typically transient and clinically insignificant in women with normal baseline lipid profiles, it may become pronounced in individuals with underlying metabolic disturbances or pre-existing dyslipidemia (29, 30).

Clinical evidence indicates that, in such cases, IVF can act as a trigger for severe hypertriglyceridemia and, consequently, acute pancreatitis. Cases of this complication have been reported over time in the literature (31–33), supporting a rare but consistent association. These observations support consideration of lipid profile assessment and TG monitoring in selected high-risk patients before and during assisted reproductive procedures.

## 6 Pregnancy in familial hypertriglyceridemia

In patients presenting with extremely high triglyceride levels, distinguishing between multifactorial chylomicronemia syndrome (MCS) and familial chylomicronemia syndrome (FCS) is of high clinical relevance. The vast majority of cases fall within the MCS spectrum, primarily driven by underlying metabolic conditions (34). These patients generally show a favorable response to dietary modifications and conventional pharmacotherapy, such as fibrates (34). In contrast, the management of FCS presents a greater therapeutic challenge and demands heightened clinical vigilance.

Familial chylomicronemia syndrome (FCS) is a rare autosomal recessive disorder characterized by markedly reduced lipoprotein lipase (LPL) activity and severe hypertriglyceridemia (often >1000 mg/dL), typically presenting early in life (35). Clinicians should strongly suspect this diagnosis when such TG elevations are accompanied by a personal history of recurrent acute pancreatitis (AP) or a family history of severe dyslipidemia or pancreatitis (36). In pregnant women with FCS, the principal concern is the high risk of AP, which can lead to recurrent pregnancy loss and poses a significant threat to maternal health.

Frequent monitoring of TG levels is recommended for women with severe hypertriglyceridemia, including FCS: every 4 weeks at the beginning of pregnancy, and every 1–2 weeks as the pregnancy progresses (8). In severe cases, hospitalization should be considered to prevent AP (8).

Management of FCS in pregnancy is challenging, as conventional lipid-lowering therapies—including fibrates, omega-3 fatty acids, niacin, and statins—often lack efficacy (37). Long-term TG control relies primarily on strict dietary fat restriction. Although dietary interventions may help reduce the incidence of AP, they are frequently insufficient to lower TG levels adequately. In such cases, lipoprotein apheresis plays a pivotal role, demonstrating reductions in TG levels of 60–90%. However, the effect is usually transient, necessitating repeated sessions every 1–2 weeks (9).

Currently, two pharmacological agents—olezarsen and volanesorsen—are approved for use in patients with FCS, both reducing TG levels by approximately 40–70%. According to the 2025 ESC/EAS guidelines, volanesorsen is indicated in patients with severe hypertriglyceridemia due to FCS, particularly in those at high risk of acute pancreatitis (38). However, data on their safety in pregnancy are limited, and they should therefore only be used in life-threatening situations when all other therapeutic options have been exhausted.

Effective management of pregnancy in women with FCS requires a multidisciplinary approach and individualized care, carefully balancing maternal and fetal risks against potential benefits.

## 7 Lipid testing during pregnancy

Currently, there is no global consensus regarding routine lipid profile screening for all pregnant women. However, emerging data suggests that undiagnosed dyslipidemia remains a significant clinical concern. A study conducted by Gowala et al., which screened 236 patients in the first trimester of pregnancy, revealed abnormal lipid profiles in 25% of the cohort (n = 59) (39). Specifically, hypertriglyceridemia (TG ≥ 150 mg/dL) was detected in 14.7% of women (n = 35). Furthermore, this screening strategy facilitated the identification of a patient potentially suffering from familial hypercholesterolemia.

Given these findings and the well-established physiological rise in lipid levels during the second and third trimesters, we propose a routine lipid profile assessment in the first and second trimester. This approach would enable the early detection of pre-existing dyslipidemia and the implementation of preventive measures against complications—such as acute pancreatitis or preeclampsia—prior to the onset of physiological gestational hyperlipidemia.

Reference ranges established by Sun et al. suggest an upper limit of normal for serum triglycerides in healthy pregnant women in the first trimester at approximately 200 mg/dL and 300 mg/dL in the second and 440 mg/dL in the third trimester) (40). However, the risk of adverse pregnancy outcomes may increase even at lower thresholds (4, 7, 28). Considering the non-invasive nature and high safety profile of dietary and lifestyle modifications, we recommend initiating these interventions early in pregnancy for patients presenting with ‘high-normal’ or mildly elevated triglyceride levels (e.g., >150mg/dL).

## 8 Therapeutic management of hypertriglyceridemia in pregnancy

Managing hypertriglyceridemia during pregnancy poses significant clinical challenges. First, there are no universally accepted reference ranges to determine when intervention is necessary. Treatment decisions must therefore be individualized, considering the patient’s overall clinical condition and the risk of complications. Second, pregnancy restricts available pharmacological options, as many drugs routinely used to lower triglycerides are contraindicated due to potential teratogenic effects or insufficient safety data. Third, severe hypertriglyceridemia in pregnancy carries substantially higher risks for both mother and fetus, necessitating prompt but carefully considered interventions.

The first trimester presents notable challenges, as this period of intense organogenesis renders the fetus highly susceptible to potential teratogenic effects of medications. A key clinical question is at what TG concentration pharmacological treatment should be initiated, especially given the elevated risk of acute pancreatitis. Safety data for omega-3 fatty acids during early pregnancy are limited, but current evidence suggests a relatively favorable profile. Supplementation with omega-3 polyunsaturated fatty acids, abundant in a Mediterranean-style diet, may also reduce maternal and placental inflammatory markers in obese pregnant women (41).

In cases of severe hypertriglyceridemia with imminent complications—particularly when acute pancreatitis is a concern—plasmapheresis may be the treatment of choice. This procedure provides a rapid and effective reduction in TG levels and has been reported to be safe during pregnancy.

### 8.1 Lifestyle and dietary interventions

In cases of mild hypertriglyceridemia during pregnancy, often not related to genetic lipid disorders, lifestyle modification represents the first-line therapeutic approach. Dietary intervention alone can reduce TG levels by an average of 50% (42). Notably, optimal maternal nutrition in the first trimester has been associated with higher PAPP-A concentrations, lower uterine artery (UtA) mean pulsatility index (PI), and decreased placental volume at first-trimester screening, indicating a lower risk of pregnancy complications such as intrauterine growth restriction (IUGR) and hypertensive disorders, including preeclampsia (43). The Mediterranean diet has been shown to promote placental and fetal growth through its anti-inflammatory and antioxidant properties (44). By reducing pro-inflammatory molecules, such as interleukins and chemokines, it promotes proper placental implantation and function, thereby lowering the risk of fetal growth restriction. Additionally, as per The American College of Obstetricians and Gynecologists (ACOG), women with risk factors, including history of preeclampsia, are advised to initiate low-dose aspirin prophylaxis (75–150 mg) before 16 weeks of gestation to reduce the risk of preeclampsia (45).

Maintaining an appropriate pregestational body weight and ensuring healthy gestational weight gain are crucial. The higher the pregestational BMI, the smaller the recommended weight gain during pregnancy. Complete avoidance of alcohol and nicotine is also strongly advised (46).

The optimal macronutrient composition of the diet in pregnant women with hypertriglyceridemia remains a subject of clinical debate.

Traditionally recommended diet is based on dietary fat restriction (<20%, or in severe cases <10% of total energy intake) (8). The rationale for fat restriction is that dietary fat is packaged into chylomicrons in the intestine; thus, limiting fat intake reduces chylomicron formation and postprandial triglyceride excursions. When implementing a low-fat diet, ensuring sufficient intake of essential omega-3 and omega-6 fatty acids, as well as fat-soluble vitamins (A, D, E, K), is essential (37).Supplementation with medium-chain triglycerides (MCTs) may be considered, as they do not elevate circulating TG levels. Care must be taken to avoid excessive caloric restriction, which could increase the risk of IUGR (47). Ideally, such interventions should be supervised by a dietitian and tailored to the individual patient (8).

Nevertheless, low-fat diet is often difficult to maintain and may inadvertently lead to increased carbohydrate intake, which can exacerbate hypertriglyceridemia through enhanced hepatic VLDL production (8).

Another possible approach is a low-carbohydrate diet. In most studies, low carbohydrate diets are defined by the carbohydrate contribution to overall energy intake, usually less than 40–45% of total energy (48). Importantly, a distinction should be made between ketogenic diets and moderate carbohydrate restriction. Strict low-carbohydrate or ketogenic diets are contraindicated during pregnancy due to the risk of maternal ketosis and its potential adverse effects on fetal development (49). However, a moderate reduction in carbohydrate intake, particularly simple sugars and high glycemic index foods, may represent a practical and effective strategy for lowering triglyceride levels. In clinical practice, such an approach may improve adherence compared to severe fat restriction. The Institute of Medicine recommends a minimum carbohydrate intake of ≥175 g/day during pregnancy (50). Accordingly, a low-carbohydrate diet in this population may also be defined pragmatically as one providing less than the currently recommended 175 g of carbohydrates per day. The precise threshold of carbohydrate intake at which a low-carbohydrate diet induces ketosis during pregnancy remains unclear. Therefore, dietary recommendations should be individualized, balancing the need to limit triglyceride-raising nutrients while ensuring adequate intake of essential fatty acids and overall nutritional sufficiency.

Beyond dietary management, physical activity is a key component of a healthy lifestyle. Exercise supports optimal body weight, blood pressure control, and facilitates the uptake of TGs by skeletal muscles during aerobic activity, lowering circulating levels. Principles of exercise during pregnancy are generally similar to those for the general population. However, a thorough clinical evaluation and review of medical history should precede any program to identify contraindications. A moderate-intensity exercise program of at least 20–30 minutes per day, most days of the week, is recommended (51). Examples of suitable activities include walking, stationary cycling, aerobic exercises, dancing, resistance training (e.g., weights, elastic bands), stretching, hydrotherapy, and water aerobics. Exercise during pregnancy has been associated with higher rates of vaginal delivery and lower incidences of excessive gestational weight gain, gestational diabetes mellitus (GDM), hypertensive disorders of pregnancy, preterm birth, cesarean delivery, and lower neonatal birth weight (52).

Stress reduction during pregnancy is also important, as it lowers the maternal cortisol/cortisone ratio, a marker associated with smaller placental volume and impaired fetal growth. Maternal stress reduces placental expression of 11β-hydroxysteroid dehydrogenase type 2 (11β-HSD2), the enzyme responsible for converting cortisol to inactive cortisone (53), restricts umbilical artery blood flow, and impairs fetal growth (54, 55). These findings underscore the importance of psychological support and psychotherapy programs aimed at improving maternal well-being during pregnancy.

### 8.2 Pharmacological interventions

In cases where lifestyle modifications fail to achieve adequate triglyceride control, or in the presence of significant hypertriglyceridemia, pharmacotherapy should be considered. The following subsections summarize the main pharmacological agents used in the management of hypertriglyceridemia. The major pharmacological and non-pharmacological interventions have been summed up in **Table 1**.

**Table 1:** Comparison of different triglyceride-lowering interventions.

Intervention	Mechanism	TG-lowering efficacy	Safety in pregnancy	Notes	Ref.
Lifestyle changes	Multiple mechanisms	~ -50%	Safe	low-fat diet + physical activity + stress reduction	(42, 43, 52, 53)
Fibrates	PPAR-α activation	~ -40%	Generally safe AFTER 1^st^ trimester	potentially increase risk of fetal malformations if used in 1^st^ trimester	(56–58)
Statins	HMG-CoA reductase inhibition	from -10% up to -40%	Potentially teratogenic	can be used in the third trimester as preeclampsia prevention	(59, 60)
Omega-3 FA	Multiple mechanisms	~ -30%	Generally safe	low to moderate doses are safe, whereas safety of high doses is not explicitly established	(61, 62, 68, 69)
Niacin	Multiple mechanisms	~ -30%	Potentially teratogenic/insufficient data		(70–72)
TPE	Extracorporeal particle removal	up to -97%	Generally safe		(90, 91, 101)
Olezarsen, Volanesorsen	Apo C-III inhibition	~ -70%	Insufficient data		(76, 78, 79)

TG, Triglyceride; FA, Fatty Acids; TPE, Therapeutic plasma Exchange.

#### 8.2.1 Fibrates

Fibrates are a class of drugs regarded as first-line therapy for hypertriglyceridemia in the general population. They act as agonists of peroxisome proliferator-activated receptor alpha (PPAR-α), which leads to reduced synthesis of fatty acids, triglycerides, and VLDL, as well as increased HDL production. As monotherapy, fibrates can lower triglyceride levels by 30–50% (56, 57).

Due to limited safety data during pregnancy, fibrates are not routinely recommended for hypertriglyceridemia in pregnant women. A retrospective cohort study in Korea did not show a statistically significant association between first-trimester fibrate exposure and the overall risk of congenital malformations (adjusted OR 1.06; 95% CI: 0.70–1.60). However, the small sample size (n = 260) limits the statistical power. Notably, in women exposed for more than 60 days in early pregnancy, a statistically significant increase in fetal malformations was observed (OR 1.32; 95% CI: 1.03–1.69) (58). These findings do not allow a definitive conclusion regarding fibrate safety, particularly with prolonged exposure. Since organogenesis occurs mainly in the first trimester, initiating fibrates during this period should be approached with caution. When clinically justified, fibrate therapy should be limited to the shortest effective duration, following a thorough risk–benefit assessment and consideration of alternative strategies.

#### 8.2.2 Statins

Statins are widely prescribed lipid-lowering agents, primarily for hypercholesterolemia. They inhibit HMG-CoA reductase, a key enzyme in cholesterol biosynthesis, and also reduce triglyceride levels by 10–40% (59). Despite their efficacy in the general population, statins are generally not recommended during the first trimester of pregnancy due to potential teratogenic effects. However, some statins, particularly pravastatin, can be safely used after 12 weeks of gestation for specific indications, such as preeclampsia prevention (60).

Statin use in pregnancy should be reserved for exceptional circumstances, such as severe familial hypercholesterolemia in high cardiovascular-risk patients, when apheresis is not available. Decisions regarding statin therapy, particularly in the first trimester, must be individualized and carried out under strict specialist supervision.

#### 8.2.3 Omega-3 fatty acids

Omega-3 fatty acids, particularly eicosapentaenoic acid (EPA) and docosahexaenoic acid (DHA), are well-established agents for the treatment of hypertriglyceridemia. Their mechanisms include reduction of hepatic lipogenesis and enhancement of fatty acid β-oxidation (61). In the general population, administration of ≥2–4 g/day (EPA + DHA) can reduce triglyceride levels by 20–40%, depending on baseline levels. For example, in the MARINE trial, treatment with 4 g/day of EPA ethyl ester led to a 33.1% reduction in triglycerides (n = 76, p < 0.0001) in patients with baseline triglycerides of 680 mg/dL, while 2 g/day resulted in a 19.7% reduction (n = 73, p = 0.0051) (62). The effect depends on both dose and formulation (ethyl esters vs. triglycerides).

During pregnancy, omega-3 fatty acids are important due to DHA’s role in fetal brain and retinal development. They may also modulate inflammation via mechanisms such as terminating neutrophil tissue infiltration, initiating phagocytosis, downregulating pro-inflammatory cytokines, and supporting tissue regeneration. These effects may contribute to improved pregnancy outcomes and prolonged gestation (63). Daily intake of 100–300 mg is generally recommended for pregnant women through diet or supplementation (64). However, higher therapeutic doses used for severe hypertriglyceridemia have limited safety data, and omega-3 fatty acids pass into breast milk (65, 66). While generally beneficial for infant development, the effects of high-dose exposure on newborns remain unclear (67). Given their favorable safety profile, omega-3 preparations should be considered a therapeutic option in pregnant women with hypertriglyceridemia (68). According to a 2024 position statement by the European Board and College of Obstetrics and Gynaecology (EBCOG) pregnant women at increased risk of preterm births due to inadequate DHA intake should receive a regular supply of about 600–1000 mg/day of DHA plus EPA, or DHA alone (69).

#### 8.2.4 Niacin

Niacin (vitamin B3, nicotinic acid) is occasionally used to treat dyslipidemia, including hypertriglyceridemia. Its primary mechanism is the inhibition of hepatic triglyceride synthesis (70). Niacin can lower triglycerides by 20–45%; however, achieving this effect requires high doses (1500–3000 mg/day) (71). Its use is limited by frequent adverse effects, including flushing, pruritus, dizziness, abnormal liver function, hyperglycemia, and hyperuricemia. Safety data for niacin during pregnancy are scarce, and it is not routinely recommended. Nonetheless, case reports describe successful niacin use in pregnancy when other therapies were ineffective (72). Initiation of niacin should be considered on an individual basis, after careful risk–benefit assessment, and under strict specialist supervision.

#### 8.2.5 Bile acid sequestrants

Bile acid sequestrants (colestipol, colesevelam, cholestyramine) are the only lipid-lowering agents approved for unrestricted use during pregnancy (73). They bind bile acids in the intestinal lumen, reducing fat emulsification and absorption. Bound bile acids are excreted, leading to increased hepatic cholesterol utilization for bile acid synthesis, which further lowers serum cholesterol levels. Their safety during pregnancy and the postpartum period results from their lack of systemic absorption. These agents are commonly used in the management of intrahepatic cholestasis of pregnancy.

However, bile acid sequestrants often fail to reduce triglyceride levels and may even increase them, particularly in patients with baseline triglycerides >400 mg/dL (74, 75). This effect may be mitigated when used in combination with fibrates, statins, or niacin. In isolated hypertriglyceridemia, bile acid sequestrants are generally considered contraindicated (75).

#### 8.2.6 Olezarsen

Several ongoing clinical trials are evaluating novel pharmacological agents designed to reduce triglyceride (TG) concentrations. One of the most promising drugs is olezarsen, which lowers TG levels by suppressing hepatic synthesis of apolipoprotein C-III. The BALANCE phase 3 clinical trial investigated olezarsen in patients with familial chylomicronemia syndrome (FCS), a rare genetic disorder characterized by markedly elevated serum TG levels and recurrent episodes of acute pancreatitis (AP). The study enrolled 66 participants randomized into three arms: olezarsen 80 mg, olezarsen 50 mg, or placebo. After six months, patients treated with olezarsen 80 mg exhibited a significant TG reduction of −43.5 percentage points compared with placebo. During the 53-week follow-up, 11 AP episodes occurred in the placebo group, compared with only 1 in the olezarsen group. Four moderate adverse events were reported among patients receiving the higher dose of olezarsen (76). Recruitment is currently ongoing for another phase 3 trial assessing the efficacy, safety, and tolerability of olezarsen in patients with severe hypertriglyceridemia (77). To date, no data are available regarding olezarsen use during pregnancy; consequently, the manufacturer does not recommend its administration in pregnant women.

#### 8.2.7 Volanesorsen

Another agent under investigation is volanesorsen, an antisense oligonucleotide that inhibits hepatic production of apolipoprotein C-III. In a phase 3 clinical trial involving patients with multifactorial severe hypertriglyceridemia or FCS, participants were randomized to receive volanesorsen or placebo. After three months of therapy, plasma TG concentrations decreased by 71.2% in the volanesorsen group compared with placebo. During follow-up, five AP episodes occurred, all in the placebo group. Reported adverse events were predominantly mild to moderate injection-site reactions. Two serious adverse events were noted: thrombocytopenia < 50,000/μL and serum sickness (78). Currently, no active clinical trials with volanesorsen are underway. Similar to olezarsen, volanesorsen should be used cautiously during pregnancy due to the lack of robust safety data; however, animal studies have not demonstrated teratogenicity or embryotoxicity.

Two published case reports have described the safe use of volanesorsen during pregnancy in women with FCS. In the first case, a 21-year-old woman who had been receiving volanesorsen since age 18 continued therapy throughout an unrecognized pregnancy until 38 weeks of gestation, when treatment was discontinued. She subsequently delivered a healthy infant, who remains in normal development at 24 months of age. In the second case, a patient discontinued volanesorsen six months prior to planned conception, which led to a marked TG increase. Despite dietary restriction and fibrate therapy initiated at 14 weeks of gestation, she developed AP at 22 weeks. Volanesorsen was reintroduced at 23 weeks, accompanied by regular therapeutic plasma exchange. No further AP episodes occurred, and she delivered a healthy infant at 35 weeks of gestation; the child remains healthy at 19 months (79). Although further studies are required to establish the safety of volanesorsen in pregnancy, these reports provide valuable clinical insight and suggest that the drug may represent a therapeutic option for selected pregnant patients with refractory hypertriglyceridemia.

#### 8.2.8 PCSK9 inhibitors

Potential future therapeutic strategies may also include lipid-lowering agents currently approved for other indications. Proprotein convertase subtilisin/kexin type 9 (PCSK9) inhibitors selectively bind to PCSK9, a protein involved in low-density lipoprotein receptor (LDLR) degradation (80). By increasing LDLR availability on hepatocytes, PCSK9 inhibitors enhance the clearance of LDL cholesterol (LDL-C) from plasma. While their principal effect is LDL-C reduction, they may also modestly lower TG levels. In the LAPLACE-2 trial, which included nearly 2,000 patients with primary hypercholesterolemia or mixed dyslipidemia, evolocumab combined with statins achieved TG reductions of 12–23% and 14–30% compared with placebo, depending on dosing. Mean percentage changes were calculated from baseline to the average of weeks 10 and 12 (81). Findings from the ODYSSEY COMBO I and II trials with alirocumab also demonstrated TG reductions, although without statistical significance (82, 83). No clinical data exist regarding PCSK9 inhibitor use in pregnant women; therefore, their administration is not currently recommended. Nevertheless, treatment decisions should be individualized based on maternal and fetal risk–benefit assessment.

#### 8.2.9 Lomitapide

Lomitapide, a microsomal triglyceride transfer protein (MTP) inhibitor, is approved for the treatment of homozygous familial hypercholesterolemia. A meta-analysis of five clinical trials, including approximately 100 patients, showed a mean TG reduction of −51.49% from baseline with lomitapide (84). However, its use during pregnancy is contraindicated due to the absence of reliable safety data.

#### 8.2.10 Inclisiran

Inclisiran, a small interfering RNA (siRNA), has also been investigated within the ORION clinical trial program. Available results have not provided conclusive evidence regarding its effects on TG concentrations. While some TG reductions have been observed, the clinical significance of this finding remains uncertain, and considerable inter-individual variability has been reported (85).

#### 8.2.11 Plozasiran

Another novel small interfering RNA (siRNA) agent is Plozasiran. It is a therapeutic targeting apolipoprotein C-III (APOC3), an important regulator of triglyceride-rich lipoprotein metabolism. By inhibiting hepatic APOC3 synthesis, plozasiran enhances lipoprotein lipase activity and promotes clearance of triglyceride-rich particles, leading to substantial reductions in serum triglyceride (TG) concentrations. In the SHASTA-2 phase 2 randomized clinical trial subcutaneously administered plozasiran achieved a significant reduction in apoC3 levels to –77% compared to placebo and a reduction of mean triglyceride level to –57% at 24 weeks (86).

Plozasiran has recently received regulatory approval in the United States for the treatment of patients with FCS or very high TG levels, representing an important advancement in the management of severe hypertriglyceridemia. However, data regarding its use during pregnancy remain extremely limited. A single case report published in 2025 provides preliminary insight into the potential use of plozasiran in the periconceptional setting. A 34-year-old woman with persistent chylomicronemia participating in the PALISADE trial received a 25mg dose of plozasiran 8 weeks prior to an unplanned conception. Treatment was discontinued upon confirmation of pregnancy and no further doses were administered. Notably, throughout gestation, triglyceride levels remained below 10 mmol/L (880 mg/dL), despite the expected physiological increase during the second and third trimesters, and no episodes of acute pancreatitis occurred. The patient delivered a healthy infant at 39 weeks of gestation. This observation suggests a prolonged triglyceride-lowering effect of plozasiran, consistent with its long hepatic half-life, and raises the possibility that preconception administration may help mitigate pregnancy-associated triglyceride elevation. However, given that evidence is limited to a single case, no definitive conclusions regarding maternal or fetal safety can be drawn (87, 88).

#### 8.2.12 Solbinsiran

Solbinsiran is a GalNAc-conjugated small interfering RNA (siRNA) targeting hepatic angiopoietin-like protein 3 (ANGPTL3), a regulator of triglyceride and lipoprotein metabolism. In the phase 2 PROLONG-ANG3 trial published in The Lancet, subcutaneous administration (days 0 and 90) in patients with mixed dyslipidemia resulted in significant and durable reductions in multiple atherogenic particles, including triglycerides (up to ~50%), LDL-C, non-HDL cholesterol, and apolipoprotein B at 6 months, with the 400 mg dose showing the most consistent efficacy. The therapy was generally well tolerated, with no major safety signals reported (89). Although solbinsiran represents a promising emerging option for combined dyslipidemia and hypertriglyceridemia, no data are currently available regarding its use in pregnancy.

### 8.3 Therapeutic apheresis

Therapeutic apheresis (TA) is an established extracorporeal procedure applied across various clinical indications. Due to the limited number of meta-analyses and randomized controlled trials, recommendations for managing severe hypertriglyceridemia (HTG) are primarily derived from observational studies and case reports. The American Society for Apheresis recommends TA both in the acute phase (strong recommendation) and for recurrence prevention (weak recommendation) of acute pancreatitis (AP) secondary to severe HTG, defined as TG > 1,000 mg/dL (>11.3 mmol/L). The principal treatment modalities include therapeutic plasma exchange (TPE) and lipoprotein apheresis (LA) (90).

TPE—often referred to as plasma exchange—involves separation of plasma from cellular blood components by centrifugation or membrane filtration, followed by removal and replacement with a substitution fluid (e.g., human albumin, plasma, or a mixture of crystalloid and colloid solutions) (90). LA selectively removes lipoprotein particles from blood while reinfusing other components. Available systems employ various physicochemical mechanisms, including filtration, precipitation, adsorption, polyclonal immunoadsorption, or ligand-based methods utilizing dextran sulphate or polyacrylate (90). Reports indicate that a single TA session can lower TG levels by 50% to as much as 97% (90–93). During TPE, TG clearance may be further enhanced through supplementation of lipoprotein lipase (LPL) deficiency by administration of fresh frozen plasma (FFP) (94).

Beyond its lipid-lowering effect, TA exerts anti-inflammatory and antithrombotic properties, improving microcirculatory perfusion through vasodilatory and hemorheological mechanisms (95–97). TA is generally considered safe. The most common complications of TPE include fever, urticaria, symptoms of hypocalcemia, and mild hypotension. In an analysis of 1,727 procedures, the frequency of adverse events varied according to the replacement fluid used: complications occurred in 42% of procedures with FFP compared with 30% when albumin and saline were used (P < 0.0001) (98). Adverse events during LA are typically related to vascular access, with transient hypotension, discomfort, chills, or hypocalcemia occasionally observed (99, 100). No adverse reactions directly attributable to blood-derived product administration have been reported.

Wind et al. indicated that the safety profile of TPE in pregnant women appears comparable to that in non-pregnant patients. However, given the potential risk of hypotension and consequent reduction in placental perfusion and fetal oxygenation, the procedure should be performed by an experienced multidisciplinary team with appropriate preparatory measures (101, 102).

Available data on HTG-related AP confirm that higher baseline TG concentrations are associated with poorer outcomes. The therapeutic goal is to reduce TG levels to the mild-to-moderate range (500–1,000 mg/dL). TA sessions are typically performed daily for one to three days, depending on the clinical course and TG response (90). Although severe HTG accounts for up to 50% of AP episodes in pregnancy (compared with approximately 4–10% in the general population), specific diagnostic and therapeutic guidelines for pregnant women are still lacking (103).

AP during pregnancy most commonly develops in the third trimester or early postpartum period and is associated with increased risk of organ failure, intensive care unit admission, preterm delivery, preeclampsia, placental abruption, and intrauterine fetal demise. Ducarme et al. demonstrated that early detection and immediate supportive therapy reduced maternal mortality from 37% to 0% and fetal mortality from 60% to 3% (104, 105). A study by Tang et al., evaluating plasma exchange versus standard therapy in Chinese patients with HTG-associated AP (including pregnant women), showed that TPE not only increased daily TG clearance and shortened the time to achieve TG < 500 mg/dL, but also significantly reduced local complications, shortened hospitalization, and decreased the rate of recurrent AP episodes (14% vs. 24% in the conservatively treated group) (106).The comparison of different triglyceride-lowering interventions has been summarized in **Table 1**. 

## 9 Treatment of acute pancreatitis associated with hypertriglyceridemia

Acute pancreatitis (AP) is a medical emergency that requires prompt intervention. Its incidence in pregnancy is estimated at 1 in 1,000–10,000 pregnancies (103). During pregnancy, the leading cause of acute pancreatitis (AP) is gallstone disease, followed by hypertriglyceridemia (HTG) (107). Less frequent causes include alcohol consumption, certain medications (e.g., azathioprine), hyperparathyroidism, infections, and trauma. In some cases the cause remains idiopathic (108, 109).

In HTG-induced AP, triglyceride levels typically exceed 1,000 mg/dL; however, the risk of AP rises linearly once TG concentrations surpass 500 mg/dL, with an estimated 4% increase in risk for each additional 100 mg/dL (110). Physiological metabolic changes during pregnancy further increase this risk, with most cases occurring in the third trimester.

Management of HTG-induced AP during pregnancy follows principles similar to those in the general population. First, adequate intravenous fluid resuscitation is essential to maintain hydration and correct electrolyte imbalances. Pain control, typically with paracetamol, is also crucial. The primary therapeutic goal, however, is rapid reduction of TG levels, with a target of <500 mg/dL (110).

**Figure 3:**
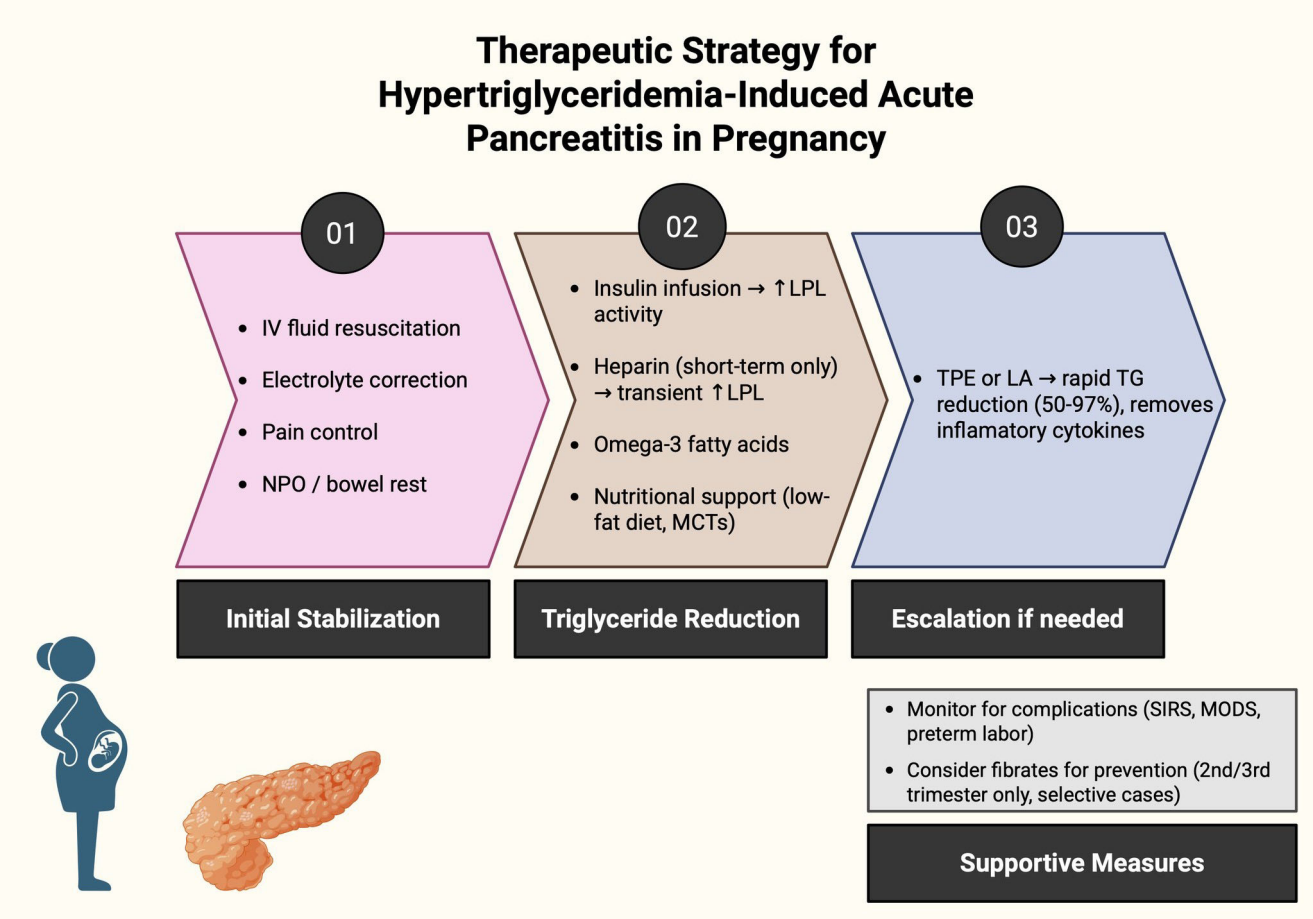
Therapeutic strategy for hypertriglyceridemia-induced acute pancreatitis in pregnancy (LPL, lipoprotein lipase; MCTs, medium-chain triglycerides; TPE, therapeutic plasma exchange; LA, lipoprotein apheresis; TG, triglycerides; SIRS, systemic inflammatory response syndrome; MODS, multiple organ dysfunction syndrome).

Several pharmacological options are available. Insulin therapy enhances lipoprotein lipase (LPL) activity, promoting chylomicron and VLDL degradation and lowering TG levels (111). Careful glucose monitoring is necessary to prevent hypoglycemia. Heparin can stimulate LPL release and reduce TG levels, but should be used short-term, as prolonged administration may deplete LPL stores, leading to rebound hypertriglyceridemia (112). Omega-3 fatty acids may also help reduce TG levels. The recommended therapeutic approach has been summarized in **Figure 3**.

In severe HTG with AP unresponsive to conservative or pharmacological treatment, plasma exchange is recommended as a second-line therapy. A single session is often sufficient, although repeated procedures may be necessary in more severe cases (113). Plasma exchange removes circulating lipoproteins and inflammatory cytokines, which is beneficial in AP complicated by systemic inflammatory response syndrome (SIRS) or multiple organ dysfunction syndrome (MODS) (9).

Historically, HTG-induced AP in pregnancy carried high maternal and fetal mortality, with reports of maternal mortality up to 20% and fetal loss up to 50% (114). Advances in diagnostics, supportive care, and neonatal management have reduced maternal mortality below 3% and fetal mortality to approximately 12% (114).

Preventive strategies are gaining attention. In selected cases, cautious use of fenofibrate may be considered in the second and third trimesters if benefits outweigh risks. Prophylactic plasma exchange has also been shown to effectively reduce the risk of AP, acting as a bridge until safe completion of pregnancy (115).

## 10 Conclusions

**Figure 4:**
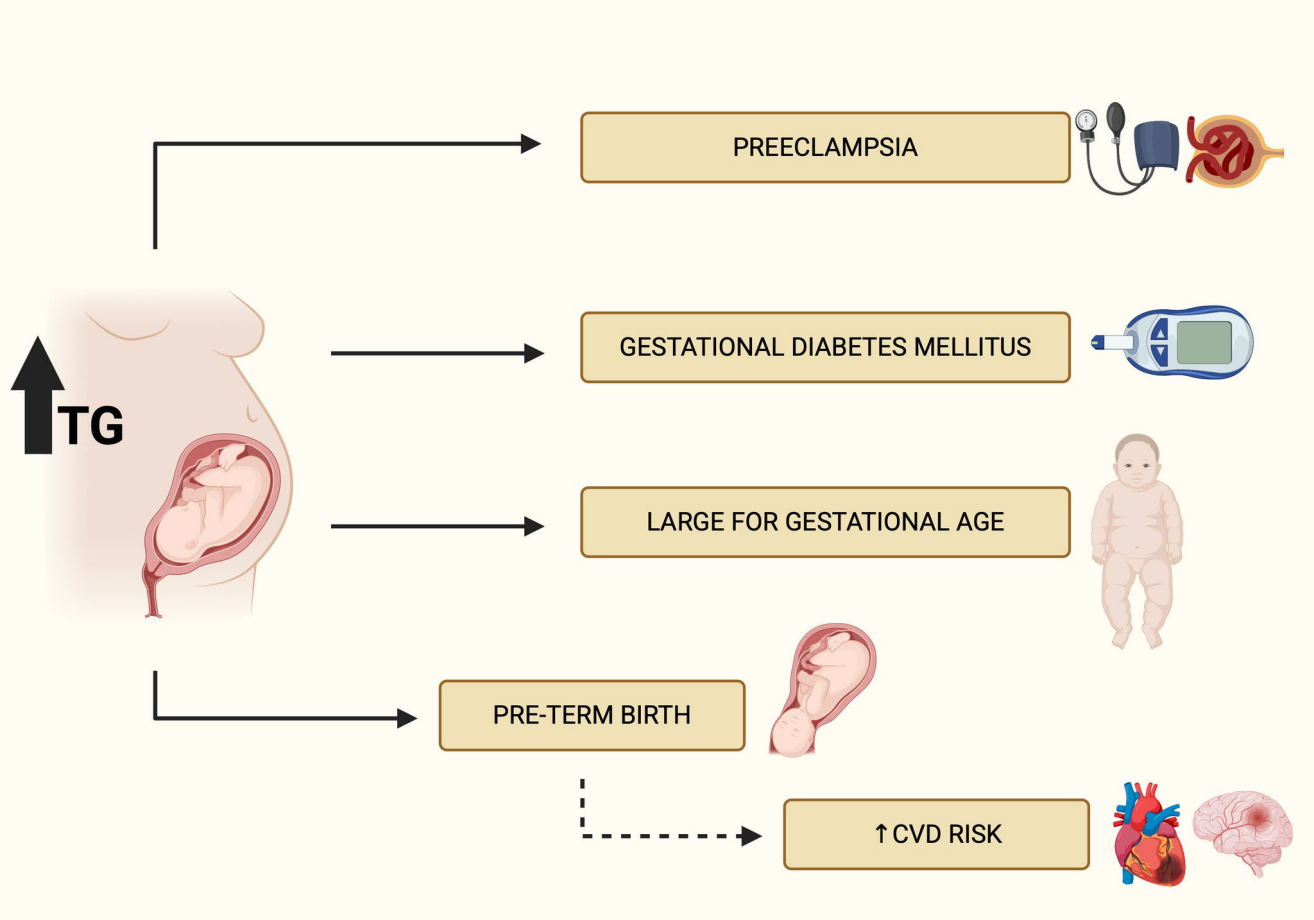
Complications of hypertriglyceridemia in pregnancy.

Hypertriglyceridemia in pregnancy represents a significant clinical challenge due to physiological metabolic adaptations and limited therapeutic options that ensure fetal safety. While elevated plasma lipid levels are a normal adaptation to support fetal and placental growth, in some patients these changes become pathological, increasing the risk of serious obstetric and metabolic complications. Notably, elevated triglyceride levels in the first trimester may serve as an early predictive biomarker for preeclampsia, gestational hypertension, gestational diabetes, preterm delivery, and abnormal neonatal birth weight. In such cases, low-dose aspirin prophylaxis may be considered, as hyperlipidemia is a recognized risk factor for hypertensive disorders of pregnancy (45, 116). Complications of hypertriglyceridemia in pregnancy have been presented in **Figure 4**.

Acute pancreatitis remains the most severe complication of hypertriglicerydemia during pregnancy, carrying a high risk of maternal and fetal mortality. Management requires prompt diagnosis and may involve intensive pharmacological therapy (e.g., insulin, heparin, omega-3 fatty acids) and extracorporeal interventions such as therapeutic plasma exchange or lipoprotein apheresis. In milder cases, lifestyle modification—including a low-fat diet, weight control, and individualized dietary guidance—is the cornerstone of therapy.

Given the lack of standardized management guidelines, the presence of comorbidities, and the limited number of safe medications during pregnancy, the care of pregnant patients with hypertriglyceridemia requires a multidisciplinary team and careful risk assessment. Considering emerging evidence on the long-term consequences of early lipid disturbances for both mother and child, future research should focus on establishing diagnostic and therapeutic standards and on monitoring the long-term cardiometabolic health of women following pregnancies complicated by hypertriglyceridemia.

## 11 Limitations

This review has several limitations. As a narrative review, it did not follow a systematic search strategy, predefined inclusion criteria, or formal quality appraisal, which may introduce selection and interpretation bias. The evidence base for hypertriglyceridemia in pregnancy is inherently limited: randomized controlled trials are scarce, and most available data derive from observational studies, small case series, or individual case reports. As a result, many management recommendations rely on indirect or low-certainty evidence. Considerable heterogeneity across studies - in diagnostic criteria, triglyceride thresholds, and reported maternal and fetal outcomes - limits comparability and prevents quantitative synthesis. In addition, safety data for lipid-lowering therapies in pregnancy remain sparse, particularly for fibrates, high-dose omega-3 fatty acids, antisense oligonucleotides, and other emerging agents. Much of the available information is extrapolated from non-pregnant populations or isolated exposures. Publication bias is also likely, especially for rare conditions such as familial chylomicronemia syndrome, where unusual or successful therapeutic cases are preferentially reported.

## References

[1] Formisano E Proietti E Perrone G Demarco V Galoppi P Stefanutti C . Characteristics, physiopathology and management of dyslipidemias in pregnancy: A narrative review. Nutrients. (2024) 16:2927. doi: 10.3390/nu16172927. PMID: 39275243 PMC11397408

[2] Wang C Kong L Yang Y Wei Y Zhu W Su R . Recommended reference values for serum lipids during early and middle pregnancy: A retrospective study from China. Lipids Health Dis. (2018) 17:246. doi: 10.1186/s12944-018-0885-3. PMID: 30382875 PMC6211477

[3] Piechota W Staszewski A . Reference ranges of lipids and apolipoproteins in pregnancy. Eur J Obstetrics Gynecology Reprod Biol. (1992) 45:27–35. doi: 10.1016/0028-2243(92)90190-A. PMID: 1618359

[4] Enquobahrie DA Williams MA Butler CL Frederick IO Miller RS Luthy DA . Maternal plasma lipid concentrations in early pregnancy and risk of preeclampsia. Am J Hypertens. (2004) 17:574–81. doi: 10.1016/j.amjhyper.2004.03.666. PMID: 15233976

[5] Packard CJ Boren J Taskinen M-R . Causes and consequences of hypertriglyceridemia. Front Endocrinol (Lausanne). (2020) 11:252. doi: 10.3389/fendo.2020.00252. PMID: 32477261 PMC7239992

[6] Sharma J McAlister J Aggarwal NR Wei J Mehta PK Quesada O . Evaluation and management of blood lipids through a woman’s life cycle. Am J Prev Cardiol. (2022) 10:100333. doi: 10.1016/j.ajpc.2022.100333. PMID: 35345879 PMC8956895

[7] Vrijkotte TGM Krukziener N Hutten BA Vollebregt KC van Eijsden M Twickler MB . Maternal lipid profile during early pregnancy and pregnancy complications and outcomes: The ABCD study. J Clin Endocrinol Metab. (2012) 97:3917–25. doi: 10.1210/jc.2012-1295. PMID: 22933545

[8] Wong B Ooi TC Keely E . Severe gestational hypertriglyceridemia: A practical approach for clinicians. Obstet Med. (2015) 8:158–67. doi: 10.1177/1753495X15594082. PMID: 27512474 PMC4935053

[9] Huang C Liu J Lu Y Fan J Wang X Liu J . Clinical features and treatment of hypertriglyceridemia‐induced acute pancreatitis during pregnancy: A retrospective study. J Clin Apher. (2016) 31:571–8. doi: 10.1002/jca.21453. PMID: 26946248

[10] Ghio A Bertolotto A Resi V Volpe L di Cianni G . Triglyceride metabolism in pregnancy. Adv Clin Chem. (2011) 55:133–53. doi: 10.1016/B978-0-12-387042-1.00007-1. PMID: 22126027

[11] Mulder JWCM Kusters DM Roeters van Lennep JE Hutten BA . Lipid metabolism during pregnancy: Consequences for mother and child. Curr Opin Lipidol. (2024) 35:133–40. doi: 10.1097/MOL.0000000000000927. PMID: 38408036 PMC11064913

[12] Manten GTR Franx A van der Hoek YY Hameeteman TM Voorbij HAM Smolders HC . Changes of plasma lipoprotein(a) during and after normal pregnancy in Caucasians. J Matern Fetal Neonatal Med. (2003) 14:91–5. doi: 10.1080/JMF.14.2.91.95. PMID: 14629088

[13] Sattar N Clark P Greer IA Shepherd J Packard CJ . Lipoprotein (a) levels in normal pregnancy and in pregnancy complicated with pre-eclampsia. Atherosclerosis. (2000) 148:407–11. doi: 10.1016/S0021-9150(99)00296-8. PMID: 10657577

[14] Zechner R Desoye G Schweditsch MO Pfeiffer KP Kostner GM . Fluctuations of plasma liproprotein-a concentrations during pregnancy and post partum. Metabolism. (1986) 35:333–6. doi: 10.1016/0026-0495(86)90150-2. PMID: 2937991

[15] Agarwala A Dixon DL Gianos E Kirkpatrick CF Michos ED Satish P . Dyslipidemia management in women of reproductive potential: An expert clinical consensus from the National Lipid Association. J Clin Lipidol. (2024) 18:e664–84. doi: 10.1016/j.jacl.2024.05.005. PMID: 38824114

[16] Magee LA Brown MA Hall DR Gupte S Hennessy A Karumanchi SA . The 2021 International Society for the Study of Hypertension in Pregnancy classification, diagnosis & management recommendations for international practice. Pregnancy Hypertens. (2022) 27:148–69. doi: 10.1016/j.preghy.2021.09.008. PMID: 35066406

[17] Qin X Ai F Zhou Q Zhang Y Yan X . Pre-eclampsia, gestational hypertension, and lipid levels during pregnancy: A systematic review and meta-analysis. Arch Gynecol Obstet. (2025) 312:385–402. doi: 10.1007/s00404-025-08052-0. PMID: 40407883 PMC12334498

[18] Gallos I Sivakumar K Kilby M Coomarasamy A Thangaratinam S Vatish M . Pre‐eclampsia is associated with, and preceded by, hypertriglyceridaemia: A meta‐analysis. BJOG. (2013) 120:1321–32. doi: 10.1111/1471-0528.12375. PMID: 23859707

[19] Shen H Liu X Chen Y He B Cheng W . Associations of lipid levels during gestation with hypertensive disorders of pregnancy and gestational diabetes mellitus: A prospective longitudinal cohort study. BMJ Open. (2016) 6:e013509. doi: 10.1136/bmjopen-2016-013509. PMID: 28011814 PMC5223699

[20] Li J Lu J Wang M Hu W Jin N Li X . Predictive value of second-trimester maternal lipid profiling in early-onset pre-eclampsia: A prospective cohort study and nomogram. Front Med (Lausanne). (2021) 8:688312. doi: 10.3389/fmed.2021.688312. PMID: 34926481 PMC8672138

[21] Lv Y Xu L He Z Liu X Guo Y . The association between pregnancy levels of blood lipids and the risk of preterm birth. Sci Rep. (2024) 14:10800. doi: 10.1038/s41598-024-61119-x. PMID: 38734779 PMC11088646

[22] Goldenberg RL Culhane JF Iams JD Romero R . Epidemiology and causes of preterm birth. Lancet. (2008) 371:75–84. doi: 10.1016/S0140-6736(08)60074-4. PMID: 18177778 PMC7134569

[23] Heida KY Velthuis BK Oudijk MA Reitsma JB Bots ML Franx A . Cardiovascular disease risk in women with a history of spontaneous preterm delivery: A systematic review and meta-analysis. Eur J Prev Cardiol. (2016) 23:253–63. doi: 10.1177/2047487314566758. PMID: 25665808

[24] Mohebi A Pathirana M Khoja A Wittwer M Lowe K Fisher D . Prevalence of metabolic syndrome among pregnant women: A systematic review and meta-analysis. Endocrine. (2025) 88:398–409. doi: 10.1007/s12020-025-04160-8. PMID: 39841354 PMC12069128

[25] Duttaroy AK Basak S . Maternal fatty acid metabolism in pregnancy and its consequences in the feto-placental development. Front Physiol. (2022) 12:787848. doi: 10.3389/fphys.2021.787848. PMID: 35126178 PMC8811195

[26] Sivan E Boden G . Free fatty acids, insulin resistance, and pregnancy. Curr Diabetes Rep. (2003) 3:319–22. doi: 10.1007/s11892-003-0024-y. PMID: 12866995

[27] Torres-Torres J Monroy-Muñoz IE Perez-Duran J Solis-Paredes JM Camacho-Martinez ZA Baca D . Cellular and molecular pathophysiology of gestational diabetes. Int J Mol Sci. (2024) 25:11641. doi: 10.3390/ijms252111641. PMID: 39519193 PMC11546748

[28] Enquobahrie DA Williams MA Qiu C Luthy DA . Early pregnancy lipid concentrations and the risk of gestational diabetes mellitus. Diabetes Res Clin Pract. (2005) 70:134–42. doi: 10.1016/j.diabres.2005.03.022. PMID: 16188575

[29] Reddy S Ahmad Z . *In vitro* fertilization and hypertriglyceridemic pancreatitis: Case report. J Clin Lipidol. (2022) 16:417–22. doi: 10.1016/j.jacl.2022.04.002. PMID: 35534371

[30] Palmisano BT Zhu L Stafford JM . Role of estrogens in the regulation of liver lipid metabolism. Adv Exp Med Biol. (2017) 1043:227–56. doi: 10.1007/978-3-319-70178-3_12. PMID: 29224098 PMC5763482

[31] Steinmetz OK Hashim E Falcone T Hemmings R Bourque J . Recurrent pancreatitis associated with *in vitro* fertilization. Obstetrics Gynecology. (1993) 81:890–2. 8469510

[32] Thanh NH Duong T Huyen NT Hai PD . *In vitro* fertilization-induced extreme hypertriglyceridemia with secondary acute pancreatitis in emergency department: A case report and literature review. Turk J Emerg Med. (2024) 24:255–8. doi: 10.4103/tjem.tjem_27_24. PMID: 39564441 PMC11573169

[33] Liu M Hu H . A case report of acute hyperlipidemic pancreatitis after blastocyst transfer and literature review. Front Med (Lausanne). (2026) 13:1698359. doi: 10.3389/fmed.2026.1698359. PMID: 41716789 PMC12913572

[34] Paquette M Bernard S Hegele RA Baass A . Chylomicronemia: Differences between familial chylomicronemia syndrome and multifactorial chylomicronemia. Atherosclerosis. (2019) 283:137–42. doi: 10.1016/j.atherosclerosis.2018.12.019. PMID: 30655019

[35] Spagnuolo CM Hegele RA . Etiology and emerging treatments for familial chylomicronemia syndrome. Expert Rev Endocrinol Metab. (2024) 19:299–306. doi: 10.1080/17446651.2024.2365787. PMID: 38866702

[36] Javed F Hegele RA Garg A Patni N Gaudet D Williams L . Familial chylomicronemia syndrome: An expert clinical review from the National Lipid Association. J Clin Lipidol. (2025) 19:382–403. doi: 10.1016/j.jacl.2025.03.013. PMID: 40234111

[37] Falko JM . Familial chylomicronemia syndrome: A clinical guide for endocrinologists. Endocrine Pract. (2018) 24:756–63. doi: 10.4158/EP-2018-0157. PMID: 30183397

[38] Mach F Koskinas KC Roeters van Lennep JE Tokgözoğlu L Badimon L Baigent C . 2025 Focused update of the 2019 ESC/EAS guidelines for the management of dyslipidaemias. Eur Heart J. (2025) 46:4359–78. doi: 10.1093/eurheartj/ehaf190. PMID: 40878289

[39] Golwala S Dolin CD Nemiroff R Soffer D Denduluri S Jacoby D . Feasibility of lipid screening during first trimester of pregnancy to identify women at risk of severe dyslipidemia. J Am Heart Assoc. (2023) 12:e028626. doi: 10.1161/JAHA.122.028626. PMID: 37183838 PMC10227310

[40] Sun L Gao B Wang M Liu Y Shan Z Teng W . The establishment of lipid profiles reference ranges during pregnancy: A systematic review and meta-analysis. Reprod Biol Endocrinol. (2025) 23:110. doi: 10.1186/s12958-025-01450-8. PMID: 40721791 PMC12302840

[41] Nakaki A Denaro E Crimella M Castellani R Vellvé K Izquierdo N . Effect of Mediterranean diet or mindfulness‐based stress reduction during pregnancy on placental volume and perfusion: A subanalysis of the <scp>IMPACT BCN</scp> randomized clinical trial. Acta Obstet Gynecol Scand. (2024) 103:2042–52. doi: 10.1111/aogs.14874. PMID: 39037192 PMC11426209

[42] Rhodes KS Weintraub M Marchlewicz EH Rubenfire M Brook RD . Medical nutrition therapy is the essential cornerstone for effective treatment of “refractory” severe hypertriglyceridemia regardless of pharmaceutical treatment: Evidence from a lipid management program. J Clin Lipidol. (2015) 9:559–67. doi: 10.1016/j.jacl.2015.03.012. PMID: 26228674

[43] Parisi F Savasi VM di Bartolo I Mandia L Cetin I . Associations between first trimester maternal nutritional score, early markers of placental function, and pregnancy outcome. Nutrients. (2020) 12:1799. doi: 10.3390/nu12061799. PMID: 32560356 PMC7353423

[44] Díaz-López A Díaz-Torres S Martín-Luján F Basora J Arija V . Prenatal adherence to the Mediterranean diet decreases the risk of having a small-for-gestational-age baby, ECLIPSES study. Sci Rep. (2022) 12:13794. doi: 10.1038/s41598-022-17957-8. PMID: 35963881 PMC9376108

[45] ACOG Committee Opinion No. 743 . Low-dose aspirin use during pregnancy. Obstetrics Gynecology. (2018) 132:e44–52. doi: 10.1097/AOG.0000000000002708. PMID: 29939940

[46] Bashir M Navti OB Ahmed B Konje JC . Hyperlipidaemia and severe hypertriglyceridaemia in pregnancy. Obstetrician Gynaecologist. (2023) 25:196–209. doi: 10.1111/tog.12887. PMID: 40046247

[47] Neha Rao SS Shantharam Baliga B Mithra P Manjrekar P Kamath N . Influencing variables for fetal growth in malnourished mothers: A nested case-control study. Clin Epidemiol Glob Health. (2020) 8:581–5. doi: 10.1016/j.cegh.2019.12.007. PMID: 38826717

[48] Tanner H Barrett HL Callaway LK Wilkinson SA Dekker Nitert M . Consumption of a low carbohydrate diet in overweight or obese pregnant women is associated with longer gestation of pregnancy. Nutrients. (2021) 13:3511. doi: 10.3390/nu13103511. PMID: 34684512 PMC8538994

[49] Tanner HL Dekker Nitert M Callaway LK Barrett HL . Ketones in pregnancy: Why is it considered necessary to avoid them and what is the evidence behind their perceived risk? Diabetes Care. (2021) 44:280–9. doi: 10.2337/dc20-2008. PMID: 33444162

[50] Institute of Medicine . Dietary Reference Intakes for Energy, Carbohydrate, Fiber, Fat, Fatty Acids, Cholesterol, Protein, and Amino Acids. Washington, D.C: National Academies Press (2005). doi: 10.17226/10490

[51] American College of Obstetricians and Gynecologists (ACOG) Committee on Obstetric Practice . Physical activity and exercise during pregnancy and the postpartum period. Obstetrics Gynecology. (2020) 135:e178–88. doi: 10.1097/AOG.0000000000003772. PMID: 32217980

[52] Berghella V Saccone G . Exercise in pregnancy! Am J Obstet Gynecol. (2017) 216:335–7. doi: 10.1016/j.ajog.2017.01.023. PMID: 28236414

[53] Glover V Bergman K Sarkar P O’Connor TG . Association between maternal and amniotic fluid cortisol is moderated by maternal anxiety. Psychoneuroendocrinology. (2009) 34:430–5. doi: 10.1016/j.psyneuen.2008.10.005. PMID: 19019559

[54] Bronson SL Bale TL . The placenta as a mediator of stress effects on neurodevelopmental reprogramming. Neuropsychopharmacology. (2016) 41:207–18. doi: 10.1038/npp.2015.231. PMID: 26250599 PMC4677129

[55] Hewitt DP Mark PJ Waddell BJ . Glucocorticoids prevent the normal increase in placental vascular endothelial growth factor expression and placental vascularity during late pregnancy in the rat. Endocrinology. (2006) 147:5568–74. doi: 10.1210/en.2006-0825. PMID: 16959835

[56] Staels B Dallongeville J Auwerx J Schoonjans K Leitersdorf E Fruchart J-C . Mechanism of action of fibrates on lipid and lipoprotein metabolism. Circulation. (1998) 98:2088–93. doi: 10.1161/01.CIR.98.19.2088. PMID: 9808609

[57] Zimetbaum P Frishman WH Kahn S . Effects of gemfibrozil and other fibric acid derivatives on blood lipids and lipoproteins. J Clin Pharmacol. (1991) 31:25–37. doi: 10.1002/j.1552-4604.1991.tb01883.x. PMID: 2045526

[58] Kay HY Jang HY Kim I-W Oh JM . Fibrates and risk of congenital malformations: a nationwide cohort study in South Korea. Arch Gynecol Obstet. (2024) 310:1967–73. doi: 10.1007/s00404-023-07357-2. PMID: 38553644 PMC11393199

[59] Stein EA Lane M Laskarzewski P . Comparison of statins in hypertriglyceridemia. Am J Cardiol. (1998) 81:66B–9B. doi: 10.1016/S0002-9149(98)00041-1. PMID: 9526817

[60] Costantine MM Cleary K Hebert MF Ahmed MS Brown LM Ren Z . Safety and pharmacokinetics of pravastatin used for the prevention of preeclampsia in high-risk pregnant women: a pilot randomized controlled trial. Am J Obstet Gynecol. (2016) 214:720.e1–720.e17. doi: 10.1016/j.ajog.2015.12.038. PMID: 26723196 PMC4884459

[61] Skulas-Ray AC Wilson PWF Harris WS Brinton EA Kris-Etherton PM Richter CK . Omega-3 fatty acids for the management of hypertriglyceridemia: a science advisory from the American Heart Association. Circulation. (2019) 140:e673–e691. doi: 10.1161/CIR.0000000000000709. PMID: 31422671

[62] Bays HE Ballantyne CM Kastelein JJ Isaacsohn JL Braeckman RA Soni PN . Eicosapentaenoic acid ethyl ester (AMR101) therapy in patients with very high triglyceride levels (from the Multi-center, placebo-controlled, randomized, double-blind, 12-week study with an open-label extension [MARINE] trial). Am J Cardiol. (2011) 108:682–90. doi: 10.1016/j.amjcard.2011.04.015. PMID: 21683321

[63] Regidor PA Eiblwieser J Steeb T Rizo JM . Omega-3 long chain fatty acids and their metabolites in pregnancy outcomes for the modulation of maternal inflammatory- associated causes of preterm delivery, chorioamnionitis and preeclampsia. F1000Res. (2024) 13:882. doi: 10.12688/f1000research.153569.3. PMID: 39931317 PMC11809487

[64] Coletta JM Bell SJ Roman AS . Omega-3 fatty acids and pregnancy. Rev Obstet Gynecol. (2010) 3:163–71. PMC3046737 21364848

[65] Valencia-Naranjo A Manjarres-Correa LM Bermúdez-Cardona J . Pilot study of the effect of EPA + DHA supplementation on the fatty acid profile of erythrocytes and breast milk of lactating women from Sonsón, Colombia. Curr Res Food Sci. (2022) 5:789–97. doi: 10.1016/j.crfs.2022.04.008. PMID: 35540308 PMC9079638

[66] Bzikowska-Jura A Czerwonogrodzka-Senczyna A Jasińska-Melon E Mojska H Olędzka G Wesołowska A . The concentration of omega-3 fatty acids in human milk is related to their habitual but not current intake. Nutrients. (2019) 11:1585. doi: 10.3390/nu11071585. PMID: 31336991 PMC6683022

[67] Nevins JEH Donovan SM Snetselaar L Dewey KG Novotny R Stang J . Omega-3 fatty acid dietary supplements consumed during pregnancy and lactation and child neurodevelopment: a systematic review. J Nutr. (2021) 151:3483–94. doi: 10.1093/jn/nxab238. PMID: 34383914 PMC8764572

[68] Bielecka-Dąbrowa A Banach M Bandosz P Burchardt P Chlebus K Dobrowolski P . Prevention of cardiovascular diseases in women planning pregnancy and during pregnancy. Expert opinion of the Section of Prevention and Epidemiology of the Polish Cardiac Society, the Polish Society of Gynecologists and Obstetricians, the Polish Society of Family Medicine, the Polish Lipid Association, and the Polish Lifestyle Medicine Society. Polish Heart J. (2025) 83:1107–23. doi: 10.33963/v.phj.107847. PMID: 40792679

[69] Savona-Ventura C Mahmood T Mukhopadhyay S Louwen F . Omega-3 fatty acid supply in pregnancy for risk reduction of preterm and early preterm birth: a position statement by the European Board and College of Obstetrics and Gynaecology (EBCOG). Eur J Obstetrics Gynecology Reprod Biol. (2024) 295:124–5. doi: 10.1016/j.ejogrb.2024.02.009. PMID: 38354604

[70] Kamanna VS Kashyap ML . Mechanism of action of niacin. Am J Cardiol. (2008) 101:S20–6. doi: 10.1016/j.amjcard.2008.02.029. PMID: 18375237

[71] Yuan G Al-Shali KZ Hegele RA . Hypertriglyceridemia: its etiology, effects and treatment. Can Med Assoc J. (2007) 176:1113–20. doi: 10.1503/cmaj.060963. PMID: 17420495 PMC1839776

[72] Suda N Leon-Martinez D Peter PR Flannery CA Irani RA . Management of severe hypertriglyceridemia in pregnancy with niacin: reevaluating safety and therapeutic benefits. Case Rep Endocrinol. (2025) 2025:2644678. doi: 10.1155/crie/2644678. PMID: 39949380 PMC11824309

[73] Lewek J Bielecka-Dąbrowa A Toth PP Banach M . Dyslipidaemia management in pregnant patients: a 2024 update. Eur Heart J Open. (2024) 4:oeae032. doi: 10.1093/ehjopen/oeae032. PMID: 38784103 PMC11114474

[74] Aldridge MA Ito MK . Colesevelam hydrochloride: a novel bile acid-binding resin. Ann Pharmacother. (2001) 35:898–907. doi: 10.1345/aph.10263. PMID: 11485143

[75] Insull W . Clinical utility of bile acid sequestrants in the treatment of dyslipidemia: a scientific review. South Med J. (2006) 99:257–73. doi: 10.1097/01.smj.0000208120.73327.db. PMID: 16553100

[76] Stroes ESG Alexander VJ Karwatowska-Prokopczuk E Hegele RA Arca M Ballantyne CM . Olezarsen, acute pancreatitis, and familial chylomicronemia syndrome. N Engl J Med. (2024) 390:1781–92. doi: 10.1056/NEJMoa2400201. PMID: 38587247

[77] Marston NA Bergmark BA Alexander VJ Prohaska TA Kang YM Moura FA . Olezarsen for managing severe hypertriglyceridemia and pancreatitis risk. N Engl J Med. (2026) 394:429–41. doi: 10.1056/NEJMoa2512761. PMID: 41211918

[78] Gouni-Berthold I Alexander VJ Yang Q Hurh E Steinhagen-Thiessen E Moriarty PM . Efficacy and safety of volanesorsen in patients with multifactorial chylomicronaemia (COMPASS): a multicentre, double-blind, randomised, placebo-controlled, phase 3 trial. Lancet Diabetes Endocrinol. (2021) 9:264–75. doi: 10.1016/S2213-8587(21)00046-2. PMID: 33798466

[79] Wanninayake S Ochoa-Ferraro A Patel K Ramachandran R Wierzbicki AS Dawson C . Two successful pregnancies -in patients taking volanesorsen for familial chylomicronemia syndrome. JIMD Rep. (2024) 65:249–54. doi: 10.1002/jmd2.12435. PMID: 38974616 PMC11224504

[80] Page MM Watts GF . PCSK9 inhibitors - mechanisms of action. Exp Clin Pharmacol. (2016) 39:164–7. doi: 10.18773/austprescr.2016.060. PMID: 27789927 PMC5079795

[81] Robinson JG Nedergaard BS Rogers WJ Fialkow J Neutel JM Ramstad D . Effect of evolocumab or ezetimibe added to moderate- or high-intensity statin therapy on LDL-C lowering in patients with hypercholesterolemia. JAMA. (2014) 311:1870. doi: 10.1001/jama.2014.4030. PMID: 24825642

[82] Kereiakes DJ Robinson JG Cannon CP Lorenzato C Pordy R Chaudhari U . Efficacy and safety of the proprotein convertase subtilisin/kexin type 9 inhibitor alirocumab among high cardiovascular risk patients on maximally tolerated statin therapy: the ODYSSEY COMBO I study. Am Heart J. (2015) 169:906–915.e13. doi: 10.1016/j.ahj.2015.03.004. PMID: 26027630

[83] Cannon CP Cariou B Blom D McKenney JM Lorenzato C Pordy R . Efficacy and safety of alirocumab in high cardiovascular risk patients with inadequately controlled hypercholesterolaemia on maximally tolerated doses of statins: the ODYSSEY COMBO II randomized controlled trial. Eur Heart J. (2015) 36:1186–94. doi: 10.1093/eurheartj/ehv028. PMID: 25687353 PMC4430683

[84] Mohammed H Nasser M Bady Z Haseeb ME Darwish M Rashed MA . Efficacy and safety of lomitapide, a microsomal triglyceride transfer protein inhibitor, in homozygous familial hypercholesterolemia: a systematic review and meta-analysis of clinical trials. Atherosclerosis. (2025) 407:119960. doi: 10.1016/j.atherosclerosis.2025.119960. PMID: 38826717

[85] Katsiki N Vrablik M Banach M Gouni-Berthold I . Inclisiran, low-density lipoprotein cholesterol and lipoprotein (a). Pharmaceuticals. (2023) 16:577. doi: 10.3390/ph16040577. PMID: 37111334 PMC10143414

[86] Gaudet D Pall D Watts GF Nicholls SJ Rosenson RS Modesto K . Plozasiran (ARO-APOC3) for severe hypertriglyceridemia. JAMA Cardiol. (2024) 9:620. doi: 10.1001/jamacardio.2024.0959. PMID: 38583092 PMC11000138

[87] Larouche M Brisson D Roy N Grenon C Poirier P Muhsin M . Course of pregnancy in a woman with familial chylomicronemia syndrome treated with plozasiran, a small interfering RNA against ApoC3. JIMD Rep. (2026) 67:e70052. doi: 10.1002/jmd2.70052. PMID: 41358055 PMC12677934

[88] Watts GF Rosenson RS Hegele RA Goldberg IJ Gallo A Mertens A . Plozasiran for managing persistent chylomicronemia and pancreatitis risk. N Engl J Med. (2025) 392:127–37. doi: 10.1056/NEJMoa2409368. PMID: 39225259

[89] Ray KK Oru E Rosenson RS Jones J Ma X Walgren J . Durability and efficacy of solbinsiran, a GalNAc-conjugated siRNA targeting ANGPTL3, in adults with mixed dyslipidaemia (PROLONG-ANG3): a double-blind, randomised, placebo-controlled, phase 2 trial. Lancet. (2025) 405:1594–607. doi: 10.1016/S0140-6736(25)00507-0. PMID: 40179932

[90] Connelly‐Smith L Alquist CR Aqui NA Hofmann JC Klingel R Onwuemene OA . Guidelines on the use of therapeutic apheresis in clinical practice – evidence‐based approach from the writing committee of the American Society for Apheresis: the ninth special issue. J Clin Apher. (2023) 38:77–278. doi: 10.1002/jca.22043. PMID: 37017433

[91] Gianos E Duell PB Toth PP Moriarty PM Thompson GR Brinton EA . Lipoprotein apheresis: utility, outcomes, and implementation in clinical practice: a scientific statement from the American Heart Association. Arterioscler Thromb Vasc Biol. (2024) 44:e304–e321. doi: 10.1161/ATV.0000000000000177. PMID: 39370995

[92] Chen Z Huang X Zhang M Han N Ning Y . Rapid reduction in triglyceride levels by therapeutic plasma exchange in patients with hypertriglyceridemic pancreatitis. J Clin Apher. (2022) 37:82–90. doi: 10.1002/jca.21954. PMID: 34846767 PMC9299693

[93] Galán Carrillo I Demelo-Rodriguez P Rodríguez Ferrero ML Anaya F . Double filtration plasmapheresis in the treatment of pancreatitis due to severe hypertriglyceridemia. J Clin Lipidol. (2015) 9:698–702. doi: 10.1016/j.jacl.2015.07.004. PMID: 26350817

[94] Stefanutti C Labbadia G Morozzi C . Severe hypertriglyceridemia‐related acute pancreatitis. Ther Apheresis Dialysis. (2013) 17:130–7. doi: 10.1111/1744-9987.12008. PMID: 23551669

[95] Wieland E Schettler V Armstrong VW . Highly effective reduction of C-reactive protein in patients with coronary heart disease by extracorporeal low density lipoprotein apheresis. Atherosclerosis. (2002) 162:187–91. doi: 10.1016/S0021-9150(01)00698-0. PMID: 11947913

[96] Julius U Siegert G Kostka H Schatz U Hohenstein B . Effects of different lipoprotein apheresis methods on serum protein levels. Atheroscler Suppl. (2015) 18:95–102. doi: 10.1016/j.atherosclerosissup.2015.02.018. PMID: 25936311

[97] Ramunni A Burzo M Vernò L Brescia P . Pleiotropic effects of LDL apheresis. Atheroscler Suppl. (2009) 10:53–5. doi: 10.1016/S1567-5688(09)71811-2. PMID: 20129375

[98] Shemin D Briggs D Greenan M . Complications of therapeutic plasma exchange: a prospective study of 1,727 procedures. J Clin Apher. (2007) 22:270–6. doi: 10.1002/jca.20143. PMID: 17722046

[99] Waldmann E Parhofer KG . Lipoprotein apheresis to treat elevated lipoprotein (a). J Lipid Res. (2016) 57:1751–7. doi: 10.1194/jlr.R056549. PMID: 26889050 PMC5036372

[100] Dittrich-Riediger J Schatz U Hohenstein B Julius U . Adverse events of lipoprotein apheresis and immunoadsorption at the apheresis center at the University Hospital Dresden. Atheroscler Suppl. (2015) 18:45–52. doi: 10.1016/j.atherosclerosissup.2015.02.007. PMID: 25936304

[101] Wind M Gaasbeek AGA Oosten LEM Rabelink TJ van Lith JMM Sueters M . Therapeutic plasma exchange in pregnancy: a literature review. Eur J Obstetrics Gynecology Reprod Biol. (2021) 260:29–36. doi: 10.1016/j.ejogrb.2021.02.027. PMID: 33713886

[102] Padmanabhan A Connelly‐Smith L Aqui N Balogun RA Klingel R Meyer E . Guidelines on the use of therapeutic apheresis in clinical practice – evidence‐based approach from the writing committee of the American Society for Apheresis: the eighth special issue. J Clin Apher. (2019) 34:171–354. doi: 10.1002/jca.21705. PMID: 31180581

[103] Cruciat G Nemeti G Goidescu I Anitan S Florian A . Hypertriglyceridemia triggered acute pancreatitis in pregnancy – diagnostic approach, management and follow-up care. Lipids Health Dis. (2020) 19:2. doi: 10.1186/s12944-019-1180-7. PMID: 31901241 PMC6942404

[104] Ducarme G Maire F Chatel P Luton D Hammel P . Acute pancreatitis during pregnancy: a review. J Perinatology. (2014) 34:87–94. doi: 10.1038/jp.2013.161. PMID: 24355941

[105] Tan SYT Teh SP Kaushik M Yong TT Durai S Tien C-C . Hypertriglyceridemia-induced pancreatitis in pregnancy: case review on the role of therapeutic plasma exchange. Endocrinol Diabetes Metab Case Rep. (2021) 2021:21–0017. doi: 10.1530/EDM-21-0017. PMID: 34013888 PMC8185538

[106] Tang S Liu Y Liu C Zhao J . Effect of plasmapheresis versus standard treatment in preventing recurrent acute pancreatitis in Chinese patients with hypertriglyceridemia. Pak J Pharm Sci. (2021) 34:1255–9. 34602397

[107] Liu P Zhao P Zhao T Yu L . Pharmacological treatment of hypertriglyceridemia-induced acute pancreatitis during pregnancy: a case report and literature review. Medicine. (2025) 104:e41810. doi: 10.1097/MD.0000000000041810. PMID: 40101052 PMC11922390

[108] Saini J Marino D Badalov N Vugelman M Tenner S . Drug-induced acute pancreatitis: an evidence-based classification (revised). Clin Transl Gastroenterol. (2023) 14:e00621. doi: 10.14309/ctg.0000000000000621. PMID: 37440319 PMC10461957

[109] Mądro A . Pancreatitis in pregnancy—comprehensive review. Int J Environ Res Public Health. (2022) 19:16179. doi: 10.3390/ijerph192316179. PMID: 36498253 PMC9737239

[110] Garg R Rustagi T . Management of hypertriglyceridemia induced acute pancreatitis. BioMed Res Int. (2018) 2018:1–12. doi: 10.1155/2018/4721357. PMID: 30148167 PMC6083537

[111] Le TQ Le HTT Tran NTT Nguyen NN Tran TT . Effectiveness of continuous intravenous insulin infusion in hypertriglyceridemia-induced acute pancreatitis of varying severity: a longitudinal study. Medicine. (2025) 104:e42674. doi: 10.1097/MD.0000000000042674. PMID: 40441219 PMC12129543

[112] Podda M Murzi V Marongiu P di Martino M de Simone B Jayant K . Effectiveness and safety of low molecular weight heparin in the management of acute pancreatitis: a systematic review and meta-analysis. World J Emergency Surg. (2024) 19:30. doi: 10.1186/s13017-024-00558-3. PMID: 39256790 PMC11385836

[113] Yeh J Chen J Chiu H . Plasmapheresis for hyperlipidemic pancreatitis. J Clin Apher. (2003) 18:181–5. doi: 10.1002/jca.10063. PMID: 14699594

[114] Hughes DL Hughes A White PB Silva MA . Acute pancreatitis in pregnancy: meta-analysis of maternal and fetal outcomes. Br J Surg. (2021) 109:12–4. doi: 10.1093/bjs/znab221. PMID: 34179950 PMC10364714

[115] Gupta M Liti B Barrett C Thompson PD Fernandez AB . Prevention and management of hypertriglyceridemia-induced acute pancreatitis during pregnancy: a systematic review. Am J Med. (2022) 135:709–14. doi: 10.1016/j.amjmed.2021.12.006. PMID: 35081380

[116] de Oliveira AA Spaans F Graton ME Stokes A Kirschenman R Quon A . Aspirin improves uterine artery function in hypercholesterolemic preeclampsia. Hypertension. (2025) 82:859–71. doi: 10.1161/HYPERTENSIONAHA.124.24435. PMID: 39936305

